# Continuity in features of anxiety and attention deficit/hyperactivity disorder in young preschool children

**DOI:** 10.1007/s00787-014-0538-7

**Published:** 2014-04-01

**Authors:** Kristin Romvig Overgaard, Heidi Aase, Svenn Torgersen, Ted Reichborn-Kjennerud, Beate Oerbeck, Anne Myhre, Pål Zeiner

**Affiliations:** 1 Division of Mental Health and Addiction, Oslo University Hospital, Pb. 4959 Nydalen, 0424 Oslo, Norway; 2 Division of Mental Health, Norwegian Institute of Public Health, Oslo, Norway; 3 Centre for Child and Adolescent Mental Health, Eastern and Southern Norway, Oslo, Norway; 4 Department of Psychology, University of Oslo, Oslo, Norway; 5 Institute of Psychiatry, University of Oslo, Oslo, Norway

**Keywords:** Anxiety, Attention deficit hyperactivity disorder, Emotional dysregulation, Longitudinal, Preschool

## Abstract

Anxiety disorders and attention deficit/hyperactivity disorder (ADHD) develop before school age, but little is known about early developmental pathways. Here we test two hypotheses: first, that early signs of anxiety and ADHD at 18 months predict symptoms of anxiety and ADHD at age 3½ years; second, that emotional dysregulation at 18 months predicts the outcome of co-occurring anxiety and ADHD at age 3½ years. The study was part of the prospective Norwegian Mother and Child Cohort Study (MoBa) at the Norwegian Institute of Public Health. The 628 participants were clinically assessed at 3½ years. Questionnaire data collected at 18 months were categorized into early behavioural scales of anxiety, ADHD, and emotional dysregulation. We investigated continuity in features of anxiety and ADHD from 18 months to 3½ years of age through logistic regression analyses. Anxiety symptoms at 3½ years were predicted by early signs of anxiety (Odds ratio (OR) = 1.41, CI = 1.15–1.73) and emotional dysregulation (OR = 1.33, CI = 1.15–1.54). ADHD symptoms at 3½ years were predicted by early signs of ADHD (OR = 1.51, CI = 1.30–1.76) and emotional dysregulation (OR = 1.31, CI = 1.13–1.51). Co-occurring anxiety and ADHD symptoms at 3½ years were predicted by early signs of anxiety (OR = 1.43, CI = 1.13–1.84), ADHD (OR = 1.30, CI = 1.11–1.54), and emotional dysregulation (OR = 1.34, CI = 1.13–1.58). We conclude that there were modest continuities in features of anxiety and ADHD through early preschool years, while emotional dysregulation at age 18 months was associated with symptoms of anxiety, ADHD, and co-occurring anxiety and ADHD at age 3½ years.

## Introduction

Anxiety disorders and attention deficit/hyperactivity disorder (ADHD) tend to have an onset at a young age and result in chronicity and impairment [1, 2], but little is known about their trajectories through early preschool years. Population studies have estimated prevalence rates to be 5 % for ADHD [3] and 15 % for anxiety disorders [4] in school-age children. Both population and clinical studies have found high co-occurrence rates of anxiety and ADHD, estimated to vary from 25 to 40 % in school children [5, 6]. Similar prevalence rates and patterns of co-occurring psychopathology have been found in preschoolers [7, 8]. In a previous paper, we showed that about one-third of preschool children with ADHD symptoms had concurrent anxiety symptoms [9].

That high occurrence and co-occurrence rates are detectable in preschoolers implies a need to investigate the predictive validity of anxiety and ADHD during the first years of life. Early intervention has been recommended for both anxiety and ADHD [10, 11]. However, further clarity is needed to help clinicians distinguish early psychiatric symptoms from normative behaviour. Longitudinal studies on young preschoolers might provide guidance.

Longitudinal studies in schoolchildren have found symptom continuity for both anxiety disorders and ADHD, although with varied subtypes at different ages [12–14]. At present, a clear understanding of the early trajectories of anxiety and ADHD is complicated by the lack of valid diagnostic criteria for preschoolers [15]. Thus, most published studies on this age group have relied entirely on questionnaire data, typically reporting broader dimensions [16], mostly on externalizing behaviour [17] and rarely on internalizing problems [18]. These studies have contributed significantly by showing that early emotional and behavioural problems are not transient, even from 1 year of age [19]. The few studies that have used diagnostic criteria confirm that emotional and disruptive disorders are moderately stable throughout the preschool period [1, 2, 20].

Research has been particularly scarce on the early course of co-occurring anxiety and ADHD [21]. Studies on older children have found evidence in support of the two disorders developing independently of one another [9, 22] and segregating separately in families [23]. However, it is not yet clear whether the two disorders have a simultaneous onset or an onset at different time points. It has been suggested that the occurrence of one of either anxiety or ADHD might influence the development of the other [24], or that common underlying factors may explain the co-occurrence. Several possible explanations for the extensive co-occurrence of mental disorders have been proposed [25]. A number of studies indicate that common liability factors play an essential role and may explain the observed co-occurrence between internalizing and externalizing disorders, including anxiety and ADHD [26, 27].

One factor that has been proposed as a possible link explaining the development of anxiety in children with ADHD is emotional dysregulation, which is conceptually similar to emotional lability [28] as well as the irritable dimension of oppositional defiant disorder ODD [29]. The concepts are similarly described and are typically expressed as intense emotions and volatile mood changes [30]. Emotional dysregulation is often hypothesized to involve some deficit in the ability to modify emotional responses as a result of underlying neurobiological dysfunctions and/or temperament traits [31]. In the present study, we use the term emotional dysregulation descriptively, and thus similarly to how emotional lability and the irritable dimension of ODD has been used in studies of older children [28, 29, 32].

Emotional dysregulation has been implicated in the development of co-occurring disorders in ADHD [33–36]. As both anxiety and ADHD have an onset at a young age, emotional dysregulation as a possible risk factor for this co-occurrence needs to be investigated at an early developmental stage.

In this prospective, longitudinal study of preschool children we set out to test two hypotheses: the first that early signs of anxiety and ADHD at 18 months would predict the outcome of anxiety and ADHD symptoms at 3½ years of age, both when occurring alone and together; the second, that emotional dysregulation at 18 months of age would predict co-occurring anxiety and ADHD symptoms at 3½ years of age.

## Methods

### Study sample

A total of 628 preschool children participated in the present study. They were recruited from the Norwegian Mother and Child Cohort Study (MoBa) at the Norwegian Institute of Public Health. The MoBa is an ongoing longitudinal study from which the mothers receive questionnaires about child behaviour at several time points, including at child age 18 and 36 months. The MoBa sample has been described previously [37, 38], and the participation rate was approximately 38.5 % (www.fhi.no/morogbarn). In brief, about 89 % of the participating mothers were ethnic Norwegian, recruited from all over Norway from 1999 to 2008, and were predominantly white Caucasians [39]. The cohort now includes 109,000 children (www.fhi.no/morogbarn). The mean household income was about 40,000 Euro, which is equal to the population mean.

The questionnaire at 36 months included a total of 11 questions about hyperactivity, impulsivity and attention problems; six items from the child behaviour checklist (CBCL/1.5–5) [40], and five items from the DSM-IV-TR criteria for ADHD [41]. To identify children at 3 years of age might be at risk of developing ADHD, children from the MoBa population with a sum score above the 90th percentile on the 11 questions on hyperactivity, impulsivity, and attention problems, as well as children whose parents reported hyperactivity as a health problem, were invited to a clinical assessment from 2007 to 2011. In addition, a comparison group of children was recruited by randomly selecting among children not included by the criteria described above. Children with severe medical conditions and children with higher scores on autistic symptoms were excluded. A total of 3,452 children were invited to participate. Of the 2,798 children with high scores/hyperactivity health problems, 1,048 (37.5 %) participated in the clinical assessments. Of the 654 children invited to the comparison group, 147 (22.5 %) participated. After consenting, parents were interviewed and participants completed a clinical assessment when the child was 36–44 months.

### Ethical approval

The Regional Committee of Ethics in Medical Research, the Data Inspectorate, the Norwegian Institute of Public Health and Oslo University Hospital approved the study. Informed written consent was obtained from the parents of the children in the study.

### Measures

#### Outcome measures at 3½ years of age

Diagnostic assessment of the child was based on the preschool age psychiatric assessment (PAPA) interview with caregiver [42]. The interview was developed for preschool children from 2 to 5 years of age and includes questions on psychiatric symptoms and impairment of these symptoms on daily functioning. Interviews were conducted by graduate students in psychology trained in administration and scoring of the interview. A specialist in clinical psychology or child psychiatry supervised the scoring of the interview. A second rater, blind to any knowledge about the child and family, rescored 79 randomly selected interviews from audio tape recordings. The average intraclass correlations (ICCs) were 0.98 for total number of ADHD symptoms, 0.99 for inattentive symptoms and 0.97 for hyperactivity–impulsivity symptoms. ICC for anxiety symptoms was 0.86.

DSM-IV-TR diagnostic measures at age 3½ years, as described elsewhere [9], were derived from parent report, with symptom presence defined as being reported in the interview. Symptoms of ADHD and anxiety that had persisted for 3 months or longer were included. The following categories were defined:

1. Anxiety symptom group (ANX) (*n* = 175) was defined by the presence of one or more DSM-IV criterion of selected anxiety disorders. The four anxiety disorder subtypes known to be most frequent in preschoolers were included: specific phobia, social anxiety, separation anxiety disorder, and generalized anxiety disorder [4, 43].
2. ADHD symptom group (*n* = 248) included all preschoolers reported to meet all, or nearly all, of the DSM-IV criteria for an ADHD subtype. When there were fewer symptoms than required to fulfil the DSM-IV diagnoses (i.e., presence of only 3–5 of at least 6 criteria for either the hyperactive/impulsive or the inattentive subtypes), impairment had to be present in order for the child to be included. Impairment was considered present if the parent reported that the child showed at least some impairment in one or more areas.
3. Co-occurring ANX and ADHD symptom group (*n* = 123) included preschoolers with symptoms as defined in both (1 and 2).
4. Controls: a total of 147 children were originally selected as controls from the MoBa population. In the present study, children with ADHD symptoms above threshold (*n* = 20) or anxiety symptoms (*n* = 45) were excluded leaving a total of 82 children in the control group.

#### Predictor variables

The following measures from the 18 months MoBa questionnaire were used:

An anxiety scale was constructed based on the child behaviour check list (CBCL/1.5–5) [40] and included the following seven items: “clings to adult or is too dependent”, “is upset when separated from care-giver”, “will not sleep alone”, “opposes to go to bed at night”, “is afraid of trying new things”, “is upset about any change to the normal routine”, and “is afraid and worried”. The items were rated on a three-point scale and higher scores reflected more anxious behaviour. The mean (*M*) score was 9.3 with a standard deviation (SD) of 2.0 (range from 3 to 21). Cronbach’s alpha was 0.60.

An ADHD scale was constructed from the emotionality activity sociability (EAS) temperament measurement scale [44] and one item from the hyperactivity subscale of the child behaviour checklist (CBCL/1.5–5) [40]. Included items were “is always on the go”, “is active as soon as wakes up”, “prefers quiet games to active games”, and “cannot sit still, is hyperactive”. The first three items were rated by mothers on a five-point scale, and the last on a three-point scale. Higher scores reflected more hyperactive–impulsive behaviour (*M* = 14.7, SD = 2.1, range from 4 to 18). Cronbach’s alpha was 0.68.

An emotional dysregulation scale was constructed by the three items: “cries easily”, “gets upset or sad easily”, and “reacts intensely when upset” on a five-point response scale from very typical to not typical. These items were taken from the emotionality subscale of the EAS temperament questionnaire [44, 45]. Higher scores reflected more emotional dysregulation (*M* = 9.3, SD = 2.2, range from 3 to 15). Cronbach’s alpha was 0.62.

We subjected the 14 predictor items of the EAS and CBCL questionnaires to a principal component analysis to ensure that the items represent the three-factor structure theoretically assumed in the present sample. The Kaiser–Meyer–Olkin (KMO) measure verified the sampling adequacy for the analysis, KMO = 0.70. Bartlett’s test of sphericity, *χ* ^2^ = 1,212.4, *p* < 0.0001. An initial analysis was run to obtain eigenvalues for each component in the data. Four components had eigenvalues over Kaiser’s criterion of 1 and in combination explained 52 % of the variance. The scree plot showed inflexions that would justify retaining both three and four components with the eigenvalue of the fourth component of only 1.1 (explaining 7 % of the variance). For the purposes of the analyses presented here, the three-factor solution was used which resulted in only one item, “clings to adult or is too dependent”, loading to both the anxiety and the emotional dysregulation factors. Omitting this item from the further analyses did not alter the main findings (see Tables 3, 4). Table 1 shows the factor loadings after rotation. Component 1 represents anxiety, component 2 ADHD, and component 3 emotional dysregulation.

**Table 1:** Summary of principal component analysis results after orthogonal rotation for the 18 months early signs of anxiety (component 1), ADHD (component 2), and emotional dysregulation (component 3) (*n* = 628)

Item	Components		
	1	2	3
Will not sleep alone	**0.72**	0.14	−0.10
Opposes to go to bed at night	**0.66**	0.20	−0.15
Is upset when separated from caregiver	**0.59**	−0.08	0.19
Is upset about any change to the normal routine	**0.46**	−0.11	0.14
Is afraid of trying new things	**0.44**	−0.22	0.17
Clings to adult or is too dependent	**0.41**	0.01	**0.48**
Is afraid and worried	**0.40**	−0.19	0.14
Is always on the go	−0.04	**0.79**	0.01
Is active as soon as wakes up	−0.06	**0.74**	0.01
Prefers quiet games than active	−0.14	**0.68**	−0.09
Can not sit still, is hyperactive	0.09	**0.54**	0.30
Gets upset or sad easily	0.05	0.04	**0.80**
Cries easily	0.14	−0.17	**0.71**
Reacts intensely when upset	0.01	0.24	**0.62**
Eigen values	2.59	2.19	1.49
% of variance	18.50	15.62	10.62

Scores >0.4 are shown in bold

The distributions of the three scales were analysed in the entire MoBa sample of more than 66,000 participants at 18 months of age. Mean scores and SDs were close to the selected control group (*n* = 82) in our study: the anxiety scale (*n* = 66,542): mean = 8.4, SD = 2.1 (control group: mean = 8.5, SD = 1.2). The ADHD scale (*n* = 66,408): mean = 13.3, SD = 2.1 (control group: mean = 13.6, SD = 2.1). The emotional dysregulation scale (*n* = 66,340), mean = 8.2, SD = 2.3 (control group: mean = 9.7, SD = 2.2).

#### Possible confounders

A disruptive behaviour scale at age 18 months was constructed based on four CBCL items [40] “hits others”, “is defiant”, “doesn’t seem to feel guilty after misbehaving”, “punishment doesn’t change his/her behaviour”. The items were rated on a three-point scale (*M* = 6.6, SD = 1.5, range from 4 to 12).

Intellectual ability (IQ) at age 3½ years was estimated from a combined score of the “Object Matrices” (non-verbal intellectual ability), and the “Vocabulary Task” (verbal intellectual ability) from the short version of the Stanford-Binet Scales [46].

### Statistical analysis

Statistical analyses were performed using the Statistical Package for the Social Science (SPSS, version 18).

A principal component analysis on the 14 predictor items of the EAS and CBCL supported a three-factor structure and is described above. Analysis of variance (ANOVA) with Bonferroni corrections was performed to compare groups at 3½ years of age on background data, and reported mean behaviour scale scores at 18 months of age. The three outcome categories at 3½ years of age: ANX, ADHD, and co-occurring ANX and ADHD (all coded 1) were investigated separately in bivariate logistic regression analyses contrasted to the control group (coded 0). In the process of fitting the model, IQ and gender were entered in the respective models, but as these covariates did not contribute significantly to any of the models, they were excluded from further analyses. In the final multivariate models early signs of anxiety, ADHD, and emotional dysregulation were entered as independent variables. In addition, we adjusted for early signs of disruptive behaviour to see if the findings of the final models were confounded by disruptive behaviour. Statistical significance was defined at the *p* < 0.05 level, and all statistical tests were two-tailed.

## Results

Table 2 shows that there were no significant differences in gender distribution, age, or IQ between the four groups at 3½ years of age.

**Table 2:** Background characteristics of the three symptom groups and controls identified and assessed at age 3½ years

	ANX (*n* = 175)	ADHD (*n* = 248)	ADHD and ANX (*n* = 123)	Controls (*n* = 82)	*χ* ^2^/*F*	Group comparisons, *p*
Male (%)	78 (45)	140 (57)	67 (55)	42 (51)	6.13	ns
Age months, mean (SD)	41.9 (1.4)	41.7 (1.3)	41.6 (1.2)	41.6 (1.2)	2.01	ns
IQ, mean (SD)	101.2 (9.6)	100.8 (9.3)	100.8 (8.6)	103.0 (8.6)	1.30	ns

Controls at age 3½ years are without symptoms of ADHD and anxiety. Age in months refers to time of clinical assessment

*ANX* anxiety symptoms, *ADHD* attention deficit/hyperactivity disorder symptoms, *SD* standard deviation, *IQ* intellectual ability

Table 3 presents mean scale scores of early signs of anxiety, ADHD, and emotional dysregulation at 18 months for the three symptom groups of co-occurring ANX and ADHD (*n* = 123), ADHD (*n* = 248), ANX (*n* = 175), and controls (*n* = 82). There were statistically significant higher mean scores of anxiety at 18 months in the ANX group and in the co-occurring ANX and ADHD group compared to the ADHD group and the controls. Similarly, there was a significantly higher ADHD mean scores in both the ADHD group and the co-occurring ANX and ADHD group compared to the ANX group and the controls at 3½ years. All the three symptom groups at 3½ years had significantly higher mean scores of emotional dysregulation at 18 months compared to controls, with no significant differences in emotional dysregulation scores between the three symptom groups.

**Table 3:** Analysis of variance (ANOVA) showing mean scale scores of early signs of anxiety, ADHD and, emotional dysregulation at 18 months of age for the three symptom groups and controls

	Groups at age 3½ years				*F*	Group comparisons, *p*
	ANX (*n* = 175)	ADHD (*n* = 248)	ADHD and ANX (*n* = 123)	Controls (*n* = 82)		
Predictors at 18 months	Mean (SD)	Mean (SD)	Mean (SD)	Mean (SD)		
Anxiety scale	9.8 (2.2)	9.0 (1.7)	9.8 (2.2)	8.5 (1.2)	12.81	3 > 2, *p* = 0.002 1, 3 > 4, 1 > 2, *p* < 0.0001
ADHD scale	14.0 (2.2)	15.3 (1.9)	14.9 (2.2)	13.6 (2.1)	18.44	3 > 1, *p* = 0.003 2, 3 > 4, 2 > 1, *p* < 0.0001
Emotional dysregulation scale	9.7 (2.2)	9.3 (2.1)	9.7 (2.2)	8.0 (2.2)	12.13	1, 2, 3 > 4, *p* < 0.0001

*SD* standard deviation, *ANX* anxiety symptoms, *ADHD* attention deficit/hyperactivity disorder symptoms at age 3½ years

Only *p* values < 0.05 are reported

In the multivariate logistic regression models early signs of anxiety, ADHD, and emotional dysregulation were entered as independent variables. All three models showed good model fit (ANX and ADHD: *χ* ^2^ = 47.79, *p* < 0.0001; Hosmer–Lemeshow *χ* ^2^ = 10.10, *p* = 0.26. ANX only: *χ* ^2^ = 43.32, *p* < 0.0001; Hosmer–Lemeshow *χ* ^2^ = 3.59, *p* = 0.89. ADHD only: *χ* ^2^ = 53.00, *p* < 0.0001; Hosmer–Lemeshow *χ* ^2^ = 4.51, *p* = 0.81). Table 4 shows that signs of anxiety at 18 months predicted the outcome of both ANX and co-occurring ANX and ADHD at 3½ years of age. Signs of ADHD at 18 months of age significantly predicted ADHD symptoms at age 3½ years of age, both when occurring with, and without ANX. Emotional dysregulation at 18 months significantly predicted all three outcomes of ANX, ADHD and co-occurring ANX and ADHD symptoms at 3½ years of age. Adjusting for early signs of disruptive behaviour in the models, did not significantly alter the predictive value of 18 months anxiety, ADHD, and emotional dysregulation in either of the models.

**Table 4:** Multivariate logistic regression analyses with early signs of anxiety, ADHD and emotional dysregulation at 18 months as predictors, and the symptom groups of ANX, ADHD, ANX and ADHD at 3½ years as outcome variables

	Groups at age 3½ years		
	ANX (*n* = 175)	ADHD (*n* = 248)	ANX and ADHD (*n* = 123)
Predictors at 18 months	OR (CI)	OR (CI)	OR (CI)
Anxiety	1.41 (1.15–1.73)**	1.08 (0.88–1.31)	1.43 (1.13–1.84) *
ADHD	1.14 (0.99–1.32)	1.51 (1.30–1.76)***	1.30 (1.11–1.54)*
Emotional dysregulation	1.33 (1.15–1.54)***	1.31 (1.13–1.51)***	1.34 (1.13–1.58) ***

Each symptom group was contrasted to controls without ADHD or anxiety symptoms in the models. CI = 95 % confidence interval for odds ratio (OR)

*ANX* anxiety symptoms, *ADHD* attention deficit/hyperactivity disorder symptoms

* *p* < 0.05

** *p* < 0.01

*** *p* < 0.001

## Discussion

The results of this prospective longitudinal study support that there is modest continuity in features of anxiety and ADHD from the age of 18 months to 3½ years, both when these symptoms occurred alone and together at age 3½ years. This was in line with our first hypothesis. Emotional dysregulation at age 18 months was found to predict the outcome of co-occurring symptoms of anxiety and ADHD at 3½ years of age, but also predicted anxiety and ADHD when one symptom cluster occurred without the other. Thus, only partial support was found for our second hypothesis.

The present study indicates that it is possible to identify early features of ADHD and anxiety disorders from the age of 18 months. This is in line with other preschool studies regarding both the continuity of internalizing and externalizing behaviour [19], and on broader behavioural and emotional categories derived from diagnostic criteria [1, 2]. The majority of earlier studies on young children have reported broader behavioural dimensions. Although they have supported that symptoms of psychopathology may manifest during early preschool age [7, 8, 21, 47], there has been little diagnostic specificity in the study of continuity of symptoms in previous studies.

Our results suggest that there might be independent pathways of anxiety and ADHD from as early as 18 months of age. By combining prospectively collected questionnaire data at 18 months with information from a diagnostic interview at 3½ years of age, the present study provides novel insight into the early trajectories of the co-occurrence of anxiety and ADHD. The findings do not suggest that one is a key factor in the development of the other, in line with previous studies on preschoolers [9], school-aged children [22], and familial association [23].

Our study could not confirm that emotional dysregulation at age 18 months only predicted the co-occurrence of anxiety and ADHD symptoms. Instead, it appeared as an unspecific risk factor for symptoms of anxiety and for ADHD, separately, and when co-occurring. Our findings were, on the one hand, in line with studies on early temperamental characteristics showing that children high on negative emotionality are at risk of developing both externalizing and internalizing disorders [31, 36, 48]. On the other hand, the irritability dimension of ODD [49, 50] which is closely related to emotional dysregulation [32], was solely associated with emotional disorders in a cross-sectional study [51]. The irritability dimension has also been found to predict future anxiety and depression in longitudinal studies [29, 52–54]. As the present study found emotional dysregulation to predict all outcomes at age 3½ years, this challenges the specificity of the irritable dimension in predicting emotional disorder during early preschool age.

Adjusting for early signs of disruptive behaviour did not significantly alter the patterns of our models. This may be due to only four items of disruptive behaviour being available. In addition, only one item (defiance) may be assumed to tap directly into ODD. Within the DSM-V, the other three items appear more closely related to conduct disorder (CD), and two more specifically from the CD specifier for “limited prosocial emotions” [41].

Good predictive validity of symptoms in preschoolers could aid clinicians in identifying children in need of intervention at a young age. Our findings suggest that caution should be exercised in predicting future psychopathology when early features of anxiety, ADHD, and/or emotional dysregulation are reported at the age of 18 months. The increase in risk was low, though statistically significant. This implies that identifying children at risk at 18 months is clinically challenging. There is a risk of over-identification and giving treatment to preschool-aged children who will not develop ADHD or anxiety disorders over time. The reason for the low increase in risk through the early preschool years might be that there are vast developmental changes in cognition, language, and emotional regulation during this period [55, 56]. This creates variability in the early development of symptomatology, and may mask an underlying continuity in child psychopathology, which in turn may explain the modest elevation of risk in the current study. Studies on older preschoolers from 3 to 6 years of age indicate a higher diagnostic continuity, although only half of the children who were diagnosed at 3 years of age fulfilled the diagnostic criteria at 6 years of age [57].

The strength of the present study is that a large group of preschoolers from a population-based cohort was clinically assessed and their parents interviewed about psychiatric symptomatology. The study design, with a sampling procedure for ADHD symptoms, increased the number of children with symptoms. Clinical studies usually have selection biases of increased comorbidity, symptom severity, and impairment, particularly for preschool children as they are seldom referred to specialist clinics [58].

Our results should be viewed in light of several limitations. The MoBa had a participation rate of about 40 % with an under-representation of risk groups such as young mothers, mothers living alone, and mothers with previous birth complications [37]. One would expect that selection biases affect population representativeness of symptom distribution, but should not affect associations between symptom clusters and associated features [37]. Although this is reassuring, some associations might be affected by biases [59]. Given the substantial attrition from MoBa to the participation in the clinical examinations, we cannot with confidence rule out the possibility that our results could be affected by sampling bias. Likewise, selection bias may be assumed from the oversampling for ADHD symptoms at 36 months. This may have increased the number of children with symptoms from different diagnostic entities, which may in turn have elevated our co-occurrence rates [60]. The sampling procedure should give caution as to the generalizability of our findings.

Both interview data and questionnaire data were based on parent report, mostly from mothers, with the possibility of report bias that has been shown to affect other studies on co-occurrence of psychiatric symptoms [60, 61].

We based our analyses on reports of psychiatric symptoms from parents only. It is likely that the diagnostic symptom groups would have been different if information from preschool teachers were included in the assessments. However, most other studies on preschool psychopathology and diagnostic entities have used parental reports only [7, 8].

Our defined symptom group included children who fulfilled, or nearly fulfilled, DSM-IV criteria for anxiety and/or ADHD. We reported symptoms that had lasted for 3 months or longer without excluding any particular items such as specific phobic symptoms. This gives a risk of having included normative behaviour, and thus elevated symptom rates. We used the duration criteria of 3 months in accordance with other preschool studies [8, 15, 57], and there is a reason to assume that this would affect the groups equally and should not diminish the results in group comparisons. However, the outcome groups at age 3½ years were not diagnoses, but strictly using DSM-IV criteria may underestimate clinically significant symptoms which might represent later onset of disorders [55]. Caution should therefore be exercised regarding the generalizability of our findings.

The MoBa measures at 18 months had only a limited number of items selected from the subscales from the CBCL [40], and the EAS temperament scale [44]. Although, researchers and specialists in clinical and developmental psychology agreed on the items that were included in the MoBa, the lack of complete subscales is a limitation in our study. As we did not have full scales, caution should be exercised in assuming that the 18-month predictors represent the full measures of temperament or psychopathology as they were originally intended. Our finding that emotional dysregulation predicted both ADHD and anxiety symptoms is contrary to studies that have reported the irritable dimension of ODD to specifically predict emotional problems [51–53]. This difference may be due to our 18-month measures for emotional dysregulation not being fully in concordance with the irritable dimension of ODD. However, the principal component analysis gave support to the three-factor solution, and internal consistencies were in accordance with earlier studies on young children [62].

Children in our control group were included only when they did not have significant symptoms of ADHD and anxiety. This may have resulted in larger differences between controls and symptom groups in our study. However, the analyses of our 18-month predictors for the entire MoBa sample of over 66,000 participants did not support an under- or overestimation of problems within our control group. This, in turn, strengthens the findings of our logistic regression models.

In conclusion, this study indicates that there is a modest continuity of anxiety and ADHD through early preschool life. Future studies should investigate these trajectories further, preferably utilizing diagnostic interviews.
